# A New Look at the Impact of Maximizing on Unhappiness: Two Competing Mediating Effects

**DOI:** 10.3389/fpsyg.2018.00066

**Published:** 2018-02-06

**Authors:** Jiaxi Peng, Jiaxi Zhang, Yan Zhang, Pinjia Gong, Bing Han, Hao Sun, Fei Cao, Danmin Miao

**Affiliations:** ^1^Department of Psychology, Chengdu University, Chengdu, China; ^2^Xi’an Research Institute of High Technology, Xi’an, China; ^3^Department of Military Medical Psychology, Fourth Military Medical University, Xi’an, China; ^4^The Management Team of Graduates, Army Logistics University of PLA, Chongqing, China

**Keywords:** maximizing, decision making style, subjective well-being, achievement motivation, regret, mediation effect, suppression effect

## Abstract

The current study aims to explore how the decision-making style of maximizing affects subjective well-being (SWB), which mainly focuses on the confirmation of the mediator role of regret and suppressing role of achievement motivation. A total of 402 Chinese undergraduate students participated in this study, in which they responded to the maximization, regret, and achievement motivation scales and SWB measures. Results suggested that maximizing significantly predicted SWB. Moreover, regret and achievement motivation (hope for success dimension) could completely mediate and suppress this effect. That is, two competing indirect pathways exist between maximizing and SWB. One pathway is through regret. Maximizing typically leads one to regret, which could negatively predict SWB. Alternatively, maximizing could lead to high levels of hope for success, which were positively correlated with SWB. Findings offered a complex method of thinking about the relationship between maximizing and SWB.

## Introduction

Neoclassical economists characterize humans as “economic men” due to their omniscient rationality. However, Simon proposed that individuals cannot obtain and consider all pieces of information when making a decision. Thus, they must choose because they are bounded by their environment and cognitive capabilities, namely bounded rationality (Simon, 1972). Maximizing and satisficing are two ends of a spectrum of the decision-making style that reflects human rationality, that is, from omniscient to bounded rationality (Schwartz et al., 2002; Cheek and Schwartz, 2016). People on either side of this dispositional continuum have been labeled satisficers and maximizers. In decision-making, maximizers strive for an optimal option that expects maximum benefit, whereas satisficers intend to determine a suitable option to achieve a satisfactory outcome (Schwartz et al., 2002). Maximizers strive to make the best choices, but they are constantly dissatisfied with their decisions. For example, Iyengar et al. (2006) determined that college graduates with high maximizing tendencies secured jobs with 20% higher starting salaries than those with low maximizing tendencies did. However, maximizers were less satisfied with the jobs they obtained compared with satisficers and experienced more negative affect throughout the job-search process. Chowdhury et al. (2009) determined that if individuals were allowed to change their original decision, maximizers were more likely to do so compared with satisficers. Similarly, Lai (2011) documented that maximizers frequently change their consumption decision, thereby leading to considerably low brand loyalty. These findings suggest that maximizers are often unsatisfied with their ultimate choice and prefer to seek numerous options at any cost. This special psychological phenomenon is called the maximization paradox (Dar-Nimrod et al., 2009).

The decision-making style of maximizing is similar to a variable trait due to its intra-timeframe stability (Thunholm, 2004; Jiang et al., 2010). Gillham et al. (unpublished) conducted four testing times in 9 months by utilizing the maximization scale. The result shows that the correlations of Timing 1 with Timings 2, 3, and 4 are 0.81, 0.82, and 0.73, respectively, thereby indicating that an individual’s maximizing tendency is relatively stable. Schwartz et al. (2002) discussed this result and proposed that individuals differ in the extent to which they satisfice or maximize. Subsequently, researchers determined that the decision-making style of maximizing has a negative correlation with positive psychological indicators, such as well-being, optimism, self-esteem, and satisfaction with life. By contrast, maximizing has a positive correlation with negative psychological indicators, such as anxiety, perfectionism, and regret (Schwartz et al., 2002; Bruine de Bruin et al., 2007; Lai, 2011; Purvis et al., 2011; Ulloa et al., 2013; Moyano-Díaz et al., 2014).

Maximizers tend to be unhappy. This premise has been explored by several preliminary studies (Schwartz et al., 2002; Roets et al., 2012; Mikkelson and Pauley, 2013). Desmeules (2002) explained that a satisficer has a considerably realistic attitude toward a possible achievement and prefers external attribution. Accordingly, satisficers are less ruminate than maximizers (Desmeules, 2002; Benjamin and Heffetz, 2012). Larsen and McKibban (2008) determined that maximizers often want to obtain more than what they already possess. Furthermore, maximizers, who target “maximization,” tend to worry about the completion of their information search and hesitate when making decisions, thereby possibly resulting in their regret about the outcome of their choice (Spunt et al., 2009; Hensher et al., 2013). Tanius et al. (2009) suggested that maximizers lack a clear standard of “best”; thus, they easily immerse in constant comparison and regret and tend to negatively evaluate what they already possess.

Regret seemed to play a key role between maximizing and happiness. Evidently, regret is a negative mood and is correlated with psychological indicators, such as anxiety, depression, and well-being (Jeffries and Konnert, 2002; Jokisaari, 2003; Roese et al., 2009). Newman et al. (2017) explored maximizing in friendship selection and determined that maximizing in selecting friends was negatively related to life satisfaction, positive affect, and self-esteem, as well as positively related to negative affect and regret. Schwartz et al. (2002) determined that regret could mediate the relationship between maximizing and psychological adaption. Diab et al. (2008) suggested that life satisfaction was affected by maximization only when an individual feels regret. Therefore, we hypothesize that maximization could negatively predict one’s subjective well-being (SWB) through the mediating effect of regret.

However, maximizing is not constantly negative. Diab et al. (2008) suggested that poor psychological adaptation and life satisfaction are not a corollary for maximizing. Lai (2010) determined that positive correlations exist between maximization and several positive psychological indicators, such as optimism, desire for consistency, internal motivation, and sense of self-efficacy. Maximizers are after optimal possibilities and discontent with what they have achieved. They also look forward to a good decision outcome and work performance. Thus, they probably enjoy higher achievement motivation compared with satisficers. Achievement motivation refers to the tendency and motivation system to overcome exterior obstacles, thereby exerting one’s ability to resolve difficulties, which is an internal power to influence an individual to do something valuable and significant as perfectly as they can (Wang and Eccles, 2013; Michou et al., 2014). Achievement motivation is developed by individuals during a social competition and is relatively stable (De Castella and Byrne, 2015). This motivation includes two substructures, namely, hope for success and fear of failure. The two types of motivation can exist separately and simultaneously; the higher hope for success and lower fear of failure meant the stronger achievement motivation (Puca, 2005; Benjamin et al., 2014a). An individual with a strong hope for success is goal-oriented, active, and likely to obtain high work performance. By contrast, an individual with a strong tendency for failure avoidance is negative and recessive. Moreover, studies show that achievement motivation can affect people’s SWB and achievement motivation is specifically positively correlated with happiness (Elliot et al., 2006). We further propose a hypothesis that maximization could significantly predict SWB through the effect of achievement motivation, thereby possibly contradicting the mediating effect of regret. That is, we hypothesized that maximization could predict SWB in two ways. Maximizing typically leads one to regret, which could negatively predict SWB. Alternatively, maximizing could lead to high levels of achievement motivation that were positively correlated with SWB. Competing ways may co-exist.

In summary, this research employed university students as respondents and attempted to analyze the relationship between the decision-making style of maximizing and SWB, verification of the mediating effects of regret, and the suppressing role of achievement motivation.

## Materials and Methods

### Participants

A total of 402 first-year undergraduate students (i.e., 219 males and 183 females) enrolled in a Comprehensive university volunteered to participate in this study for additional course credits or ¥15 (∼2.5 dollars) as compensation. The age range of the participants is from 17 to 20 with a mean of 18.73 (*SD* = 1.12). The participants majored in English, Management, Philosophy, Computer Science, Geography, Environmental Engineering, and Optics. All of them provided their written informed consent before completing the measures. Thereafter, they completed the questionnaires in a classroom environment using a pencil-and-paper format. We distributed 402 inventories and collected 395 valid inventories with a valid recovery rate of 98.26%.

### Instruments

#### Maximization Scale

Schwartz et al. (2002) developed the 13-item Maximization Scale, which measures the tendency to maximize. Sample items from the scale included “I treat relationships like clothing: I expect to try a lot on before finding the perfect fit” and “When I watch TV, I channel surf, often scanning through the available options even while attempting to watch one program.” The participants indicated their degree of agreement to these descriptions using five-point scales from “1” (completely disagree) to “5” (completely agree). The Cronbach’s alpha coefficient of the Maximization Scale in our study was 0.71.

#### Regret Scale

Schwartz et al. (2002) developed the five-item Regret Scale, which measures the tendency to be regretful after making decisions (e.g., “Whenever I make a choice, I try to get information about how the other alternatives turned out”). The responses were measured on a scale anchored at “1” (completely disagree) to “5” (completely agree). The Cronbach’s alpha coefficient of the Regret Scale in our study was 0.76.

#### Achievement Motivation Scale

The achievement motivation scale (AMS) is a 30-item self-evaluation scale, which comprises two sub-scales that measure hope for success (e.g., “I feel pleasure at working on tasks that are fairly difficult for me”) and fear of failure (e.g., “I become anxious when I meet a problem I don’t understand at once”). Each item was rated using a Likert-like scale from “1” (not at all true of me) to “4” (very true of me) (Li et al., 2015). The Cronbach’s alpha coefficients of the two subscales were 0.79 and 0.70 in our study.

### Subjective Well-Being

The SWB scale was developed by Diener (2000) and comprised three sub-scales that measure life satisfaction and positive and negative affects. The life satisfaction scale includes five items, including “In most ways my life is close to my ideal” and “I am satisfied with my life,” and was rated using a seven-point scale from 1 (strongly disagree) to 7 (strongly agree). The positive and negative affect scales comprised six and eight words, respectively. Each scale describes one type of positive or negative emotion, such as “angry,” “shameful,” and “proud.” The participants were asked to rate how often they were in these emotional state using a seven-point rating scale from “1” (not at all) to “7” (all the time) (Zhang et al., 2014). The Cronbach’s alpha coefficients of the three subscales in our study were 0.82, 0.73, and 0.86, respectively.

### Mediation and Suppression Analyses

The current study analyzed the mediating and suppressing effects and used an analytic approach. We let X, M, and Y be the independent, third, and dependent variables, respectively, and Y = cX + e1, M = aX + e2, and Y = c’X + bM + e3. The mediating effect means that the impact of the independent variable on the dependent variable is achieved partially or completely through a mediator. After introducing the mediator into the regression model, the regression coefficient of X on Y would be decreased, that is, c > c’. Suppressing effect is defined when M increases the regression coefficient between X and Y by its inclusion in the regression equation, that is, c < c’ (Preacher and Hayes, 2004; MacKinnon et al., 2007). The current study hypothesized that regret is the mediator and achievement motivation is the suppressor between maximizing and SWB.

Correlation and regression analyses were performed using SPSS 16.0. Structural modeling analysis was performed using AMOS 16.0.

## Results

**Table 1** shows that maximizing was positively correlated with regret (*r* = 0.45, *p* < 0.01), hope for success (*r* = 0.13, *p* < 0.05), and negative affect (*r* = 0.21, *p* < 0.01) and negatively correlated with life satisfaction (*r* = -0.24, *p* < 0.01) and positive affect (*r* = -0.22, *p* < 0.01). In addition, regret and hope for success were correlated with all components of SWB (i.e., life satisfaction and positive and negative affects).

**Table 1:** Correlations analysis.

	1	2	3	4	5	6	7
(1) Maximizing	1						
(2) Regret	0.45**	1					
(3) Hope for success	0.13*	–0.15**	1				
(4) Fear of failure	0.08	–0.08	–0.15**	1			
(5) Life satisfaction	–0.24**	–0.50**	0.21**	0.13*	1		
(6) Positive affect	–0.22**	–0.40**	0.19**	0.07	0.61**	1	
(7) Negative affect	0.21**	0.33**	–0.11*	–0.16**	–0.42**	–0.24**	1
Mean	38.33	20	37.97	31.03	20.32	23.06	17.04
*SD*	7.79	4.30	5.66	6.84	5.11	6.59	4.17

*^∗^*p* < 0.05; ^∗∗^*p* < 0.01.*

No significant correlation exists between maximizing and fear of failure (*r* = 0.08, *p* > 0.05). This study focused on the mechanism underlying the correlation between maximizing and SWB Thus, fear of failure was excluded in the further analysis.

Following the procedure introduced by Anderson and Gerbing (1988), after correlation analysis of all observed variables, in the further analysis, the latent variable, SWB, was seen as a whole. Confirmatory factor analysis showed that all the factor loadings for the indicators (life satisfaction, positive affect, and negative affect) on the latent construct (SWB) were significant (*p* < 0.01), indicating that SWB was well represented by their indicators. Then we followed the mediation test rules introduced by Baron and Kenny (1986) and preliminarily tested the mediating role of regret and the suppressing role of achievement motivation (hope for success) between maximizing and SWB. First, **Table 2** shows that regret and hope for success were considered dependent and maximizing was regarded as independent (see Models 1 and 2), thereby revealing that the regression coefficients were all significant. Thereafter, SWB were regarded as the dependent. In model 3, maximizing significantly predicts SWB. However, in model 4, when regret was introduced as independent, the regression coefficient of maximizing to SWB became insignificant (β = -0.03, *p* = 0.50), indicating that the effect of maximizing on SWB was completely mediated by regret. In model 5, when hope for success was added, the standardized regression coefficient of maximizing to SWB increased from -0.27 to -0.32, indicating that hope for success suppressed the effect of maximizing on SWB. In model 6, it was observed that when hope for success and regret were both added, maximizing couldn’t significantly predict SWB (β = -0.08, *p* = 0.10).

**Table 2:** Multiple regression analysis.

Model	Dependent	Predictors	*B*	*SE*	*β*	*t*
1	Regret	Maximizing	0.25	0.03	0.45	10.08**
2	Hope for success	Maximizing	0.09	0.04	0.13	2.56*
3	SWB	Maximizing	–0.16	0.03	–0.27	–5.14**
4	SWB	Maximizing	–0.02	0.03	–0.03	–0.67
		Regret	–0.59	0.05	–0.56	–11.58**
5	SWB	Maximizing	–0.19	0.03	–0.32	–6.09**
		Hope for success	0.23	0.04	0.28	5.40**
6	SWB	Maximizing	–0.04	0.03	–0.08	–1.64
		Regret	–0.54	0.05	–0.53	–10.81**
		Hope for success	0.14	0.04	0.18	3.76**

**FIGURE 1:**
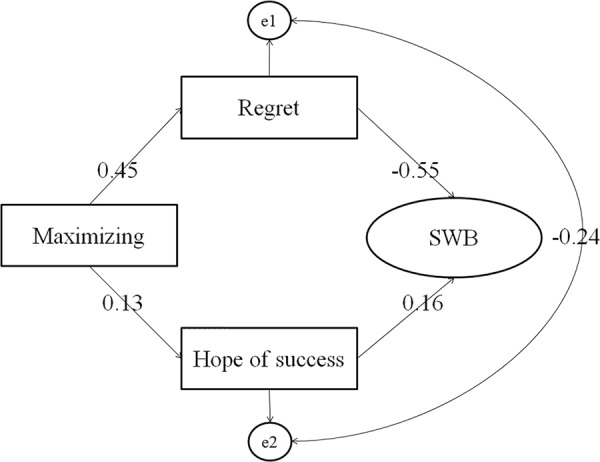
Model C presentation. All path coefficients were standardized and significant at level of *p* < 0.05.

**FIGURE 2:**
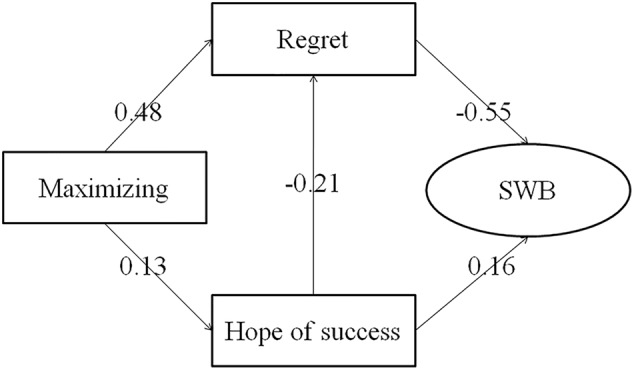
Model D presentation.

At last, the structural modeling analysis (SEM) was used to fully present the structural model. Firstly, model A containing mediators (regret and hope for success) and a direct path from maximizing to SWB was analyzed, which showed the direct path from maximizing to SWB was not significant (β = -0.08, *p* = 0.16). The model B was built deleting the direct path from maximizing to SWB. The results showed that the model not very good fit to the data, see **Table 3**. However, examination of parameter estimates can be found that the standardized path coefficient from maximizing to regret, from maximizing to hope for success, from hope for success to SWB, and from regret to SWB were all significant. Thus, according to the modification indices in the model B, model C was created by by adding the correlations of residual terms between hope for success and regret. After adding the correlations of the residual terms, the model 3 as shown in **Figure 1**, was analyzed, and it showed a good fit to the data. Correlation analysis showed that regret and hope for success was significantly correlated, and it was logical to hypothesize that those have less desire to success, may tend to fear of failure, and became overcautious and indecisive, and easy to get regret; whereas those have strong desire to success, tend to have high decision making confidence and perseverance (Dane and Pratt, 2007), and may have less regret. Additionally, achievement motivation is a stable and trait like variable, while regret is a kind of negative affect, and it was logical to hypothesize that a trait like variable could predict an affect variable. Though there were few studies and theories concerning the relation of regret and achievement motivation, model D was built by adding a path from hope to success to regret based on model B, see **Figure 2**. According to the fitness coefficients, model C and model D were considered preponderant, which both showed that two indirect paths of maximizing on SWB: indirect paths through regret and hope for success. These two paths are contradictory.

**Table 3:** Modeling fitness.

*Model*	*χ^2^/df*	CFI	RMSEA	NFI	SRMR
A	4.97	0.95	0.10	0.93	0.06
B	4.59	0.94	0.09	0.93	0.07
C	2.01	0.99	0.05	0.97	0.03
D	2.01	0.99	0.05	0.97	0.03

## Discussion

The results show that maximizing is negatively correlated with SWB, that is, a maximizer is not as happy as a satisficer. This result confirms the findings of Schwartz et al. (2002) and several previous studies. Maximizers strive for maximal benefit and “best” choice, but they are likely to be disappointed because the standard of optimal outcome is consistently uncertain (Tanius et al., 2009; Huber et al., 2012). That is, even maximizers themselves have no idea what can be called “optimal.” Their judgment standards are merely through continuous analysis of all potential options. A maximizer tends to be dependent upon social comparisons (Schwartz, 1992; Linde and Sonnemans, 2012). Schwartz et al. (2002) compared satisficers and maximizers and determined that the latter tends to have more negative affect in an upward comparison and attain less positive affect and self-enhancement in a downward comparison. An old Chinese proverb said that “happiness lies in the contentment”; however, maximizers seem difficult to satisfy. These conditions may explain why a maximizer is not as happy as a satisficer.

The results also show that regret can mediate the influence of maximizing upon SWB. This view is consistent with the view of Schwartz et al. (2002), Diab et al. (2008), Roets et al. (2012), and other scholars that regret plays an important role in maximizing and psychological inadaptability. Regret often accompanies disappointment caused by an individual’s imagination that a better outcome will occur if a discrepancy between previous and actual actions exist (Polasky et al., 2011; Zhao et al., 2015). Satisficers have clear standards for a decision outcome. Once their outcomes are achieved, they will not feel regretful (Caplin et al., 2011). By contrast, maximizers constantly compare and evaluate because they lack a clear goal (Tanius et al., 2009). When maximizers realize a better option, they will be regretful for their original choice and judgment. Regret itself is a cognitive habit for negative mood and inadaptation (O’Connor et al., 2012). Regretful individuals are often anxious and self-critical (Fisher and Exline, 2010; Monforton et al., 2012). Correspondingly, their life satisfaction is low (Gahm et al., 2010; Purvis et al., 2011). Thus, regret can mediate the effect of maximizing on SWB.

Moreover, the current study determined that the dimension of hope for success in achievement motivation can suppress the effect of maximizing on SWB. Maximizers establish a high standard for decision-making and judgment. They enhance such pursuit for optimal life and work, thereby expecting an outstanding outcome (Iyengar et al., 2006; Benjamin et al., 2014b). Maslow’s hierarchical theory of needs states that human beings use the need of self-fulfillment as basis to create an internal motivation to fulfill his goal, such as achievement motivation, thereby propelling an individual to meet the need of self-actualization by means of various activities (Taormina and Gao, 2013). Carver (2006) suggested that under the influence of achievement motivation, a positive feeling is produced when a task was accomplished by an individual. Maximizing has a strong hope for success, which is positively correlated with SWB. The finding on hope for success is related to the recent research on maximizing and eudaimonic well-being (Kokkoris, 2016), thereby exhibiting certain similarities with the idea of enjoying by striving to overcome difficulties. Thus, the influence from maximizing to SWB through achievement motivation was positive, which was opposite with indirect effect though regret. However, the latter path is dominant; hence, maximizing is still negatively correlated with SWB. In addition, no significant correlation exists between maximizing and fear of failure, which was inconsistent with our expectations. Accordingly, maximizing is the desire for the best and obtaining the best results is a type of success, although maximizing does not predict one’s attitude on failure. On the one hand, obtaining the maximum benefit takes time, patience and numerous trial-and–error undertakings. On the other hand, failure means the result farther from “best.” There lies a contradictory between the two statements, but maximizing cannot predict fear of failure. The main innovation of the current study is to determine the positive indirect effect of maximizing on SWB. Elliot and Thrash distinguished approach and avoidance temperament as basic dimensions of personality and motivational systems (Elliot, 2006; Elliot and Thrash, 2010). The former facilitated behavior and often generated positive affect, whereas the latter inhibited behavior and generated negative affect. Maximizing, which is constantly searching for the best option, seems to be considerably related to approach temperament. However, maximizing often relates to negative affect and unhappiness. Thus, we believe that maximizing indicates high standards and desire for success, which should not be maladaptive. The important degree of difference lies between simply having high standards and actually desiring the best, thereby confirming the saying that “Going too far is as bad as not going far enough.”

Additionally, this study found that hope for success and regret was negatively correlated, and hope for success could significantly predict regret. Hope for success is a positive and trait like variable and regret is a kind of negative affect. It was not difficult to understand these two variables were negatively correlated. Previous studies found that people with higher achievement motivation, would have stronger decision making confidence, and more tend to take risks under uncertainty (Collins et al., 2004; Stewart and Roth, 2007; Teychenne et al., 2015). Regret, to some extent, means not satisfied and confident with one’s own decisions, and previous studies also found that people who regret easily tend to avoid risk (Chorus, 2014). Though we found a significant path from hope for success to regret in the SEM analysis, it should be cautious to expand this finding. Some more questions remained unexplored. For example, why faire to failure didn’t significantly correlate with regret? Future studies are encouraged to further investigate the relation of achievement motivation and regret.

The current study determined two competing indirect pathways between maximizing and SWB. One pathway is through regret. Maximizing typically leads one to regret, which could negatively predict SWB. Alternatively, maximizing could lead to substantial levels of hope for success, which were positively correlated with SWB. These findings offered a considerably complex explanation of the relationship between maximizing and SWB. Nevertheless, this research also has several limitations. Most importantly, the maximization scale used in the current study is critiqued by several researchers (e.g., Diab et al., 2008; Nenkov et al., 2008). The measure of maximizing has constantly been one of the major difficult problems for researchers in this field (Cheek and Schwartz, 2016). Diab et al. (2008) criticized the maximizing scale of Schwartz et al. (2002) for failing to disentangle maximizing from maladaptive decision-making, thereby possibly misunderstanding the relationship between maximizing and happiness. Thereafter, they developed the maximizing tendency scale. In addition, uncertainty still exists on whether maximizing is a multidimensional or single-dimensional concept. Originally, Schwartz et al. (2002) did not present a clear structure of maximizing. Diab et al. (2008) supported the single-dimension model that maximizing is a general tendency to pursue the identification of an optimal alternative. Meanwhile, Nenkov et al. (2008) conducted a factor analysis of Schwartz et al.’s (2002) maximizing scale and claimed that maximizing includes the three dimensions of decision difficulty, alternative search, and high standard. Nenkov et al. (2008) also determined that a few items in the original scale do not perform well based on the internal, external, and judgmental criteria and developed three short versions of the scale (i.e., 9-, 6-, and 3-items). However, Nenkov et al. (2008) also reported that the correlations among the three dimensions and other psychological indicators are unstable. For example, the correlation coefficient of life satisfaction and alternative search was significant when using the original 13- and 6-item scales but not significant when using the 9-item scale. Cheek and Schwartz (2016) proposed a two-component model of maximization, namely, goal (selecting the best) and strategy (alternative search). Given the lack of a complete agreement among measurement experts to assess maximizing; hence, the traditional method of the measurement of maximization was adopted and maximizing was regarded as a single-dimension variable, which is similar to what many other researchers did (Parker et al., 2007; Polman, 2010). We acknowledge this method as conservative. Nevertheless, detaching maximizing with decision difficulty will clarify the relationship between maximizing and SWB. Second, the majority of the studies on maximizing have been conducted in Western countries and several cross-cultural differences in maximizing have been determined. For example, Roets et al. (2012) determined that maximizing was significantly related with well-being in Western society, but this correlativity was insignificant in non-Western society. Yang (2010) determined that in the Chinese vision of maximizing scale, the Cronbach’s alpha coefficient for the entire scale was acceptable (nearly 0.7), but that in a few dimensions was relatively low (below 0.5 for high standers dimension). In our preliminary study, we determined that a few items that Nenkov et al. (2008) thought to be inappropriate have high factor loadings in the Chinese vision. This situation is also the reason why we just calculated an entire score for maximizing. These results suggested that maximizing is a concept with cultural attribute and it is important to see this culture difference. Third, our research employed university students as participants; thus, whether our findings can be applied to other group needs further study. In addition, our research is not experimental; thus, only a correlation among variables can be concluded and not a definite causality. The explanation for the current findings should be given with caution. Fourth, from the correlations and the path coefficients in the SEM analysis, it’s apparent the mediating effect of regret is more dominant than the suppressing effect of hope for success. However, considering the controversy about effect size of mediating effect (like K2 and R2) (Preacher and Kelley, 2011), we are not comfortable comparing the effect size of mediating effect and suppressing effect. Future studies are encouraged to do this, which seems quite interesting. And it was also worthy of comparing these effect size differences when different maximizing scales are used.

## Ethics Statement

The Medical Ethics Committee of Fourth Military Medical University approved the study. Prior to testing, we obtained written consent from all participants.

## Author Contributions

Conceived and designed the experiments: JP and DM. Data collection: JP, YZ, PG, HS, and FC. Analyzed the data: JP, JZ, and BH. Contributed reagents/materials/analysis tools: JP, JZ, and DM. Wrote the manuscript: JP and DM.

## Conflict of Interest Statement

The authors declare that the research was conducted in the absence of any commercial or financial relationships that could be construed as a potential conflict of interest.
